# Non-pharmacological Methods of Deep Vein Thrombosis Prophylaxis in Orthopaedic Wards

**DOI:** 10.7759/cureus.73255

**Published:** 2024-11-07

**Authors:** Mukesh O Phalak, Ajinkya K Chaudhari, Sagar Gurnani

**Affiliations:** 1 Department of Orthopaedics, Dr. D. Y. Patil Medical College, Hospital & Research Centre, Dr. D. Y. Patil Vidyapeeth (Deemed to be University), Pune, IND

**Keywords:** deep vein thrombosis (dvt), dvt prophylaxis, post-operative management, post-operative physiotherapy, post-operative physiotherapy rehabilitation

## Abstract

Deep vein thrombosis (DVT) is a dreaded post-operative complication for surgeons and warrants prophylactic measures. The risk of DVT is significantly higher in almost any and all major surgery. The common prophylactic measures are pharmacological methods like subcutaneous low-molecular-weight heparin and oral anticoagulants. In some cases, these are contraindicated due to the risk of post-operative bleeding and wound soakage. In the editorial, we discuss the non-pharmacological methods of DVT prophylaxis in the form of basic physiotherapy. In our orthopaedic wards, we initiate basic physiotherapy like static hamstring, quadriceps exercises and ankle pumps. These exercises work by preventing the pooling of blood in the lower limbs, thereby hypothesized to prevent DVT. We have been implementing this method in all patients; hence, it has a universal application. It is cost-effective and does not have any adverse reactions. However, its use has some limitations, such as in patients with lower limb ankle fractures, polytrauma patients who are intubated, spinal cord injury patients with power loss and patients with neurological injury, although this cohort is a smaller fraction of the patients undergoing orthopaedic surgical intervention. In our experience, this method is an excellent non-pharmacological method of DVT prophylaxis which is easy to implement, is universally applicable, does not require any major special instrumentation or infrastructure and is cost-effective for the patient as well as the hospital.

## Editorial

Pharmacological prophylaxis for deep vein thrombosis (DVT) or venous thromboembolism is effective and should be considered in patients undergoing major elective surgeries to reduce the burden of DVT on healthcare providers [1]. We would like to share our experience in orthopaedic wards regarding DVT in orthopaedics encompassing a variety of patients with trauma, arthroplasty, osteomyelitis, arthroscopy, sports injuries, etc. DVT is an important complication in Indian healthcare and should be managed appropriately to avoid deterioration of the patient’s condition and unfavourable prognosis. It is commonly seen in long bone fractures like the femoral shaft, intertrochanteric femur fractures, hip and knee arthroplasties and pelvic fractures. Additional factors like geriatric age, bed-ridden status, obesity, medical comorbidities and history of DVT put the patient at a high risk for such a complication. After a major surgery, surgeons initiate pharmacological prophylaxis with either subcutaneous low-molecular-weight heparin or oral anticoagulation to mitigate DVT risk [2,3]. There are other non-pharmacological interventions like mechanical methods, such as DVT compression stockings, and physical methods, such as static hamstring, quadriceps exercises, ankle pumps and limb elevation.

We align with the thought process of taking preventive steps for tackling DVT. In our practice as a standard protocol, we initiate basic physiotherapy like ankle pumps, static hamstring and quadriceps exercises as early as possible. In most of the cases, these exercises are started on the day of the surgery once the anaesthesia effects have worn off. The patients are encouraged to start active ankle pumps and are counselled regarding the importance of the exercises and the risk of DVT if they do not comply with the instructions. If the patient cannot perform active ankle pumps due to pain or apprehension, the attendants are encouraged to perform ankle movements passively. Though active ankle pumps are more effective than passive movements, passive ankle pumps also increase the blood flow velocity in the femoral vein [4]. These basic exercises work by preventing the pooling of venous blood in the lower limbs and increasing the venous return using the gastrocsoleus complex as a secondary pump, which reduces the risk of DVT [5]. It is a non-pharmacological method and does not have any adverse effects. The cost burden of hospitalization is also reduced by avoiding use of costly drugs and mechanical devices.

These basic exercises can be utilized in most of the cases, barring a few like ankle fractures, spinal cord injuries and intubated cases of polytrauma wherein other methods like mechanical and pharmacological prophylaxis need to be implemented. The advantage of this method is that it does not affect the coagulation cascade, thereby not increasing the risk of bleeding or drain output in post-operative patients. The limitations of this method are poor compliance and lesser efficacy compared to medical methods. Also, the literature does not directly compare the efficacy of this method against mechanical and pharmacological methods. However, our experience of using basic physiotherapy exercises to prevent DVT is excellent, offering advantages like cost benefits, applicability in all patients, bare minimum adverse effects and observed reduction in the incidence of DVT. We plan to undertake a study to document the reduction in the incidence of DVT because of early initiation of basic physiotherapy exercises.
